# Effectiveness of Start to Run, a 6-week training program for novice runners, on increasing health-enhancing physical activity: a controlled study

**DOI:** 10.1186/1471-2458-13-697

**Published:** 2013-07-31

**Authors:** Linda Ooms, Cindy Veenhof, Dinny H de Bakker

**Affiliations:** 1 Netherlands Institute for Health Services Research (NIVEL), PO Box 1568, 3500 BN, Utrecht, The Netherlands; 2 Scientific Center for Transformation in Care and Welfare (Tranzo), Tilburg University, PO Box 90153, 5000 LE, Tilburg, The Netherlands

**Keywords:** Sports setting, Sporting organizations, Running, Health-enhancing physical activity, Controlled study, Follow-up

## Abstract

**Background:**

The use of the organized sports sector as a setting for health-promotion is a relatively new strategy. In the past few years, different countries have been investing resources in the organized sports sector for promoting health-enhancing physical activity. In the Netherlands, National Sports Federations were funded to develop and implement “easily accessible” sporting programs, aimed at the least active population groups. Start to Run, a 6-week training program for novice runners, developed by the Dutch Athletics Organization, is one of these programs. In this study, the effects of Start to Run on health-enhancing physical activity were investigated.

**Methods:**

Physical activity levels of Start to Run participants were assessed by means of the *S*hort *QU*estionnaire to *AS*sess *H*ealth-enhancing physical activity (SQUASH) at baseline, immediately after completing the program and six months after baseline. A control group, matched for age and sex, was assessed at baseline and after six months. Compliance with the Dutch physical activity guidelines was the primary outcome measure. Secondary outcome measures were the total time spent in physical activity and the time spent in each physical activity intensity category and domain. Changes in physical activity within groups were tested with paired t-tests and McNemar tests. Changes between groups were examined with multiple linear and logistic regression analyses.

**Results:**

In the Start to Run group, the percentage of people who met the Dutch Norm for Health-enhancing Physical Activity, Fit-norm and Combi-norm increased significantly, both in the short- and longer-term. In the control group, no significant changes in physical activity were observed. When comparing results between groups, significantly more Start to Run participants compared with control group participants were meeting the Fit-norm and Combi-norm after six months. The differences in physical activity between groups in favor of the Start to Run group could be explained by an increase in the time spent in vigorous-intensity activities and sports activities.

**Conclusions:**

Start to Run positively influences levels of health-enhancing physical activity of participants, both in the short- and longer-term. Based on these results, the use of the organized sports sector as a setting to promote health-enhancing physical activity seems promising.

## Background

The positive effects of physical activity on health and mortality have been well established. Participation in regular physical activity decreases the risk of coronary heart disease, stroke, type 2 diabetes mellitus, certain cancers (e.g. breast cancer, colon cancer), osteoporosis, obesity and falls [1-7]. Moreover, there is some evidence that physical activity is positively associated with mental health and quality of life [8,9].

Given the numerous health benefits of physical activity participation, various guidelines have been published on the recommended volume and intensity of physical activity for healthy adults. Commonly used guidelines are those developed by the American College of Sports Medicine (ACSM) and the American Heart Association (AHA). To promote and maintain health, the ACSM and AHA recommend that: “All healthy adults aged 18 to 65 years need moderate-intensity aerobic (endurance) physical activity for a minimum of 30 minutes on at least five days each week or vigorous-intensity aerobic physical activity for a minimum of 20 minutes on at least three days each week. Also, combinations of moderate- and vigorous-intensity activity can be performed to meet this recommendation.” [10] Similar guidelines have been adopted in the Netherlands and are referred to as the Dutch Norm for Health-enhancing Physical Activity (DNHPA) and the Fit-norm. Someone who meets at least one of the two guidelines adheres to the so-called “Combi-norm”, the third norm used in the Netherlands (see Table 1) [11].

**Table 1:** Dutch physical activity guidelines for adults

**Norm**	**Description**
**Dutch Norm for Health-enhancing Physical Activity (DNHPA)**	*Adults (18-54 years):*
	Thirty minutes or more of at least moderate-intensity aerobic (endurance) physical activity (≥ 4 MET; combined intensity score SQUASH ≥ 3) on at least five days each week.
	*Adults (55 years and older):*
	Thirty minutes or more of at least moderate-intensity aerobic (endurance) physical activity (≥ 3 MET; combined intensity score SQUASH ≥ 3) on at least five days each week.
**Fit-norm**	*Adults (18-54 years):*
	Twenty minutes or more of vigorous-intensity physical activity (≥ 6.5 MET; combined intensity score SQUASH ≥ 6) on at least three days each week.
	*Adults (55 years and older):*
	Twenty minutes or more of vigorous-intensity physical activity (≥ 5 MET; combined intensity score SQUASH ≥ 6) on at least three days each week.
**Combi-norm**	Meeting at least one of the previous mentioned norms (i.e. the DNHPA or Fit-norm).

Despite the existence of these guidelines, more than one third of the Dutch adult population does not engage in sufficient physical activity: in 2009, 58% of the Dutch adult population met the DNHPA, 33% met the Fit-norm, and 62% met the Combi-norm [11].

One of the ways of being physically active is through organized sports. There is large potential for the organized sports sector as a setting in which to promote health-enhancing physical activity to the general population, given the large numbers of participants, the extent of community reach and the availability of many different sports and professional trainers. Moreover, physical activity opportunities are provided on a continuous basis (i.e. people can play sport on a weekly basis at a sports club). This is in contrast with physical activity interventions, which are mostly of short or limited duration. In this way, the organized sports sector can also play an important role in maintaining physical activity levels. Another positive aspect of the organized sports setting is the possibility to socially interact with other people. As social support has been identified as a determinant of physical activity [12-14], participation in organized sports may lead to greater physical activity benefits than other forms of physical activity. It is, for example, well known that people who are involved in (organized) sports are significantly more likely to meet physical activity guidelines than those people who are not [11].

However, there are still people who are doing sports activities below the recommend levels of physical activity (i.e. with regard to frequency, duration and/or intensity) and there are also people who never play sports at all. According to recent data, 56% of the Dutch population plays sports at least once a week. For the European Union countries combined this percentage is only 40% [15]. This shows the importance of further increasing participation rates in (organized) sports.

Sports promotion has a long history in many countries, but the use of the organized sports sector as a setting to gain control over health issues and unhealthy behaviors, like physical inactivity, is a relatively new strategy [16-19]. This settings-based health promotion approach is based on the idea that changes in people’s health and health behavior are easier to achieve if health promoters focus on settings instead of individuals. It has also been applied to other settings, like schools and workplaces [20]. The approach builds on the Ottawa Charter of 1986 that stated: “Health is created and lived by people within the settings of their everyday life; where they learn, work, play and love.” [21]

In the past few years, different countries have been investing resources in the organized sports sector for promoting health-enhancing physical activity: in Australia, for example, State Sporting Associations were funded to develop healthy (e.g. smoke-free settings) and welcoming environments in their associated clubs, to ultimately increase participation in sport for health benefits [16,18]. In the Netherlands, the Dutch Ministry of Health, Welfare and Sport initiated the National Action Plan for Sport and Exercise (NAPSE). This program was aimed at increasing the number of Dutch people meeting physical activity guidelines [17]. Within the NAPSE, National Sports Federations were funded to develop and implement sporting programs tailored to the needs and abilities of the least active population groups, i.e. making sports activities easily accessible and creating a welcoming sports environment for these target groups. A total of fourteen “easily accessible” sporting programs were developed and implemented in different locations in the Netherlands. Start to Run, a 6-week training program for novice runners, developed by the Dutch Athletics Organization, is one of these programs. Participants are given the opportunity to become acquainted with the different aspects of running. Afterwards, they are stimulated to continue running as member of a local athletics club or the Dutch Athletics organization.

Running is a feasible form of a vigorous-intensity physical activity; it is not time consuming, it can be done anywhere and at any time, and only a pair of running shoes is needed. As a result, running is a popular way to become physically active, and there are many different training programs for novice runners available. There is strong literature on the health benefits of running in general and different studies have been published about (the prevention of) running related injuries [e.g. [22-26]. So far, no studies have been conducted, however, about the effectiveness of running programs on increasing health-enhancing physical activity levels. In general, there is a lack of research and evaluation of activities conducted in sports settings. Improvements in the research in this area are desirable. Particularly, there is a need for controlled study designs, incorporating both the short- and longer-term effects of sporting programs and activities, to move towards providing evidence-based programs [27,28].

Therefore, the aim of this study was to assess the effectiveness of Start to Run on increasing health-enhancing physical activity, both in the short- and longer-term, and in comparison with a control group. The results of the current study will contribute to the knowledge base concerning the effectiveness of programs initiated in sports settings, and will, consequently, provide further insight into the role of the organized sports sector in promoting health-enhancing physical activity. The study findings may be of interest to policy makers in the areas of sports and health. Also, sporting organizations may use the results when developing and implementing similar sporting programs.

## Methods

### Study design

To assess the effectiveness of Start to Run on increasing health-enhancing physical activity, a controlled study design was used. The study was performed according to Dutch legislation on privacy. The privacy regulations of the study were approved by the Dutch Data Protection Authority. According to Dutch legislation, approval by a medical ethics committee was not obligatory, as participants were not subjected to procedures, nor were they required to follow rules of behavior (i.e. participants were approached for the study after they had voluntarily registered for the Start to Run training program).

### Study population

#### Start to Run participants

Start to Run is aimed at adult novice runners who want to learn to run continuously for at least three kilometers. The program is offered two times a year (in March and September) by athletics clubs and running stores in more than hundred different locations in the Netherlands. Participants are recruited locally using different recruitment strategies (e.g. by advertisements in local media, posters, and flyers). For this study, the Dutch Athletics Organization provided data (i.e. name, email address, sex and age) of 513 individuals who had registered for the Start to Run program in March 2009. These individuals were sent an email with information about the study and a link to an online baseline questionnaire. By completing the baseline questionnaire, the Start to Run participants gave consent for participation in the study.

#### Control group participants

The control group consisted of members of the Dutch Health Care Consumer Panel of the Netherlands Institute for Health Services Research (NIVEL). This panel contains about three thousand individuals aged 18 years and older and is representative for the Dutch population with regard to age and sex. The panel members are questioned four times a year about health care, health insurance, and other related issues [29]. For the current study, 1328 panel members were approached. Control group participants did not receive any intervention. Moreover, they were asked if they had participated in the Start to Run program or any of the other NAPSE sporting programs before or during the study period, as this could influence results. Subsequently, control group members who had done so were excluded from the study. Compared with the Start to Run group, the control group members were significantly older, and were more likely to be male. As physical activity levels differ by age and sex [15,30], the control group was matched with the Start to Run group on age and sex.

### Start to Run program

During the 6-week training period, except for the last week, participants trained three times a week: one time in a group under guidance of one or more professional coaches (i.e. one coach per 15 participants), and two times individually. As a rule, training days were followed by rest days. In the last week, participants could test their running abilities in a three kilometers test run. Participation in this run, however, was not obligatory. A guided training session lasted approximately 90 minutes and consisted of a theoretical part (20-30 minutes), followed by a practical part (60-70 minutes). During the theoretical part one of the following theory items was discussed: health benefits of running and (prevention of) running-related injuries, running clothes and shoes, proper food and drinks (before, during and after training), physiological changes during running and training with a heart rate monitor. The practical part consisted of a warming-up, a run-walk part and a cooling-down. Participants were instructed to walk and perform light (stretching) exercises to warm up and to cool down. During the warming-up also attention was paid to running technique (e.g. proper posture, stride, foot strike, breathing) and running technique exercises. The run-walk part consisted of a combination of running and walking, whereby running time and distance were gradually increased during the training period. On average there were 35 participants per group session, guided by two professional coaches. An individual training session lasted approximately 45 minutes and consisted, just as the practical part of the group sessions, of a warming-up, a run-walk part and a cooling-down. Participants received instructions (e.g. training schedule, running tips) for the individual training sessions during the group sessions from their coach(es) and through weekly emails from the Dutch Athletics Organization. After completing the program, participants were stimulated to continue running. Participants were personally informed by their coach(es) about membership from this or other local athletics clubs. Additionally, participants received several emails from the Dutch Athletics Organization with information about local athletics clubs and an individual runner membership of the Dutch Athletics Organization.

### Outcome measures

Demographic data were collected for each participant, including age and sex. The level of physical activity was assessed by the *S*hort *QU*estionnaire to *AS*sess *H*ealth-enhancing physical activity (SQUASH). This instrument has proven to be fairly reliable and reasonably valid in ordering subjects according to their level of physical activity in an adult population [31]. The SQUASH measures the amount of physical activity for five domains: commuting activities, leisure-time activities, sports activities, household activities, and activities at work and school. It consists of three main queries, namely days per week, average time per day, and self-reported intensity (light, moderate or vigorous). An average week in the past month was taken as reference period. Using the Ainsworth Compendium of Physical Activities, a metabolic equivalent (MET) value was assigned to all physical activities [32]. Based on age and assigned MET values, physical activities were subdivided into three intensity categories: light, moderate and vigorous. For adults aged 18-54 years, the following cut-off values were used: < 4.0 MET (light), 4.0 to 6.5 MET (moderate), ≥ 6.5 MET (vigorous). For adults aged ≥ 55 years, the cut-off values were: < 3.0 MET (light), 3.0 to 5.0 MET (moderate), ≥ 5.0 MET (vigorous). This MET category was combined with self-reported intensity for each activity, resulting in a combined intensity score ranging from 1 to 9, with 1 being light MET and light self-reported intensity to 9 being vigorous MET and vigorous self-reported intensity. The classification of physical activities according to the combined intensity score was as follows: < 3 (light), 3 to 6 (moderate), ≥ 6 (vigorous). Subsequently, the following outcome measures were calculated: compliance with the Dutch physical activity guidelines (see Table 1); minutes per week spent in light-, moderate- and vigorous-intensity activities; minutes per week spent in commuting activities, leisure-time activities, sports activities, household activities, and activities at work and school; and total minutes per week spent in physical activity. Compliance with the Dutch physical activity guidelines was seen as the primary outcome measure, as these guidelines specify the amount of physical activity necessary to obtain health benefits. The other physical activity outcome measures were used to explain possible changes in physical activity behavior in more detail.

Start to Run participants were assessed by means of an online questionnaire at baseline (t = 0), immediately after completing the program (t = 6 weeks) and six months after baseline (t = 6 months: i.e. 4.5 months after cessation of the Start to Run training program). Control group participants were assessed at the start of the study (t = 0) by means of a postal questionnaire and six months later (t = 6 months) by means of a postal or an online questionnaire. The assessments of the control group were performed in the same months as the assessments of the Start to Run group. To increase response rates, reminders were sent one week (for online questionnaires) or two weeks (for postal questionnaires) later.

### Sample size

The sample size was based on detecting a difference in habitual physical activity according to the Fit-norm. As running is a vigorous-intensity activity, it was expected that the Start to Run program would mostly affect the percentage of people who met the Fit-norm. To detect a 20% difference between the Start to Run group and the control group six months after baseline, with a two-sided 5% significance level and a power of 80%, a sample size of 89 participants per group was necessary. Given the sample size of both the Start to Run group (n=513) and the control group (n=1328), it was expected that sufficient participants were included in the study.

### Statistical analysis

All statistical analyses were performed using Stata statistical software version 10.1 (Stata Corporation, College Station, Texas). Descriptive statistics were used to describe the main characteristics of each group and to explore baseline comparability. Means and standard deviations were calculated for continuous measures, while percentages were calculated for dichotomous measures. Differences between groups with regard to age and sex were tested with an independent t-test and chi-squared test, respectively. Changes in physical activity within groups were examined with paired t-tests for continuous physical activity measures and McNemar tests for dichotomous physical activity measures. To compare changes in physical activity between groups, multiple regression analyses (linear regression was used for continuous measures and logistic regression was used for dichotomous measures) were performed with physical activity level at six months as the dependent variable and group (Start to Run group versus control group, with the control group as the reference category) as the independent variable. Adjustments were made for baseline physical activity levels, by using this variable as a covariate in the regression model. To check if the results of the continuous physical activity outcome measures were influenced by outliers, also more robust regression techniques were applied: these techniques included the use of robust standard errors (i.e. Huber-White robust estimates of the standard errors and bootstrap estimates of the standard errors). As these robust regression techniques did not yield different results and conclusions, these results will not be presented here. P-values less than 0.05 were considered statistically significant.

## Results

### Study participants

**Figure 1:**
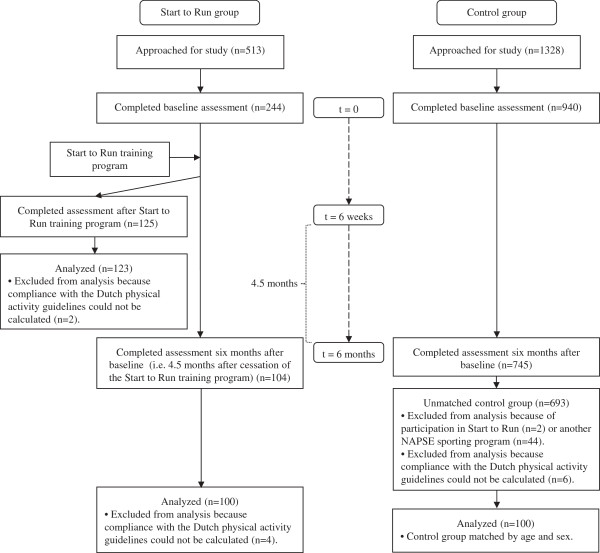
Flow of participants through the study.

The flow of participants through the study is shown in Figure 1.

#### Start to Run participants

Of 513 persons approached, 244 completed the baseline assessment. Of these 244 persons, 125 completed the assessment at six weeks. Two persons were excluded from analysis, because compliance with the Dutch physical activity guidelines could not be calculated. Therefore, data of 123 persons were available to evaluate changes in physical activity after six weeks. All persons who completed the baseline assessment (n=244) were also approached for the assessment at six months, irrespective if they had completed the assessment at six weeks. This was done to get an optimal response for comparisons with the control group. Of 244 persons approached, 104 completed the assessment at six months. Subsequently, four persons were excluded from analysis, because compliance with the Dutch physical activity guidelines could not be calculated. Consequently, data of 100 persons were available to evaluate changes in physical activity after six months and to make comparisons with the control group. There were 78 Start to Run participants who completed all three assessments (not shown in Figure 1). However, to optimally use data and maintain study power (i.e. for comparisons with the control group a sample size of 89 participants per group was necessary), all available cases were included in the analyses. This means that analyses were performed on 123 and 100 Start to Run participants for effects after six weeks and six months, respectively. Non-response analyses revealed that Start to Run participants who did not complete the assessment after six months were significantly younger (37 ± 9 years vs. 40 ± 10 years) and were more likely to be female (92.4% female vs. 70.0% female) compared with those who did complete this assessment. There were no significant differences in baseline physical activity levels between respondents and non-respondents.

#### Control group participants

Of 1328 persons approached, 940 completed the baseline assessment. Of these 940 persons, 745 completed the assessment at six months. Subsequently, 46 persons were excluded from analysis due to participation in the Start to Run program (n=2) or any of the other NAPSE sporting programs (n=44). In addition, six other persons were excluded, because compliance with the Dutch physical activity guidelines could not be calculated. Of the remaining 693 persons, 100 were matched to the Start to Run group on age and sex.

### Baseline characteristics of study participants

The baseline characteristics of the Start to Run group (i.e. the participants who completed the six months assessment) and the control group are shown in Table 2. The Start to Run participants had a mean age of 40 years (SD=10) and the control group participants had a mean age of 42 years (SD=9). The percentage of women was 70.0% in both groups. There were no significant differences in age and sex between groups. Matching was therefore successful. With regard to baseline physical activity levels, the Start to Run participants spent significantly less time in moderate-intensity physical activities (213 ± 453 min/week vs. 406 ± 596 min/week, p=0.01) and household activities (552 ± 780 min/week vs. 919 ± 968 min/week, p=0.004) compared with controls. For the remaining physical activity outcome measures, no significant differences were found between groups at baseline.

**Table 2:** Baseline characteristics of the Start to Run group and control group

	**Start to Run group**^**a**^	**Control group**	**P**
Sample size (n)	100	100	
Age (years)			
Mean ± SD	40 ± 10	42 ± 9	0.12
Min-max	21-71	23-77	
Sex (%)			
Male	30.0	30.0	1.0
Female	70.0	70.0	
Dutch physical activity guidelines (%)			
Compliance with DNHPA	48.0	59.0	0.12
Compliance with Fit-norm	56.0	55.0	0.89
Compliance with Combi-norm	58.0	65.0	0.31
Physical activity by intensity, mean ± SD (min/week)			
Light-intensity activities	1814 ± 1224	1958 ± 1263	0.42
Moderate-intensity activities	213 ± 453	406 ± 596	0.01*
Vigorous-intensity activities	238 ± 250	253 ± 337	0.73
Physical activity by domain, mean ± SD (min/week)			
Commuting activities	88 ± 137	117 ± 251	0.30
Leisure-time activities	257 ± 296	328 ± 407	0.16
Sports activities	126 ± 166	107 ± 147	0.40
Household activities	552 ± 780	919 ± 968	0.004*
Activities at work and school	1309 ± 935	1182 ± 951	0.35
Total time spent in physical activity, mean ± SD (min/week)	2265 ± 1251	2616 ± 1356	0.06

^a^Start to Run participants who completed the six months assessment.

*Significant (p<0.05) difference between groups.

### Changes in physical activity

#### Changes in physical activity after six weeks

In Table 3, physical activity outcome measures are presented for the Start to Run group at baseline and after six weeks. At baseline, 43.9% of the Start to Run participants met the DNHPA, 53.7% met the Fit-norm, and 57.7% met the Combi-norm. After six weeks, these percentages increased significantly (p<0.0001) to 74.8%, 87.0%, and 91.1% for the DNHPA, Fit-norm, and Combi-norm, respectively. Although more Start to Run participants met physical activity guidelines after six weeks, the total time spent in physical activity did not change significantly (2237 ± 1183 min/week vs. 1996 ± 1451 min/week, p=0.08). However, there were significant changes in physical activity behavior within physical activity intensity categories and domains, i.e. after six weeks, the Start to Run participants spent more time in vigorous-intensity activities (200 ± 205 min/week vs. 410 ± 298 min/week, p<0.0001), commuting activities (70 ± 110 min/week vs. 98 ± 155 min/week, p=0.01), leisure-time activities (240 ± 268 min/week vs. 301 ± 343 min/week, p=0.02) and sports activities (101 ± 143 min/week vs. 243 ± 173 min/week, p<0.0001), while less time was spent in light-intensity activities (1827 ± 1192 min/week vs. 1423 ± 1296 min/week, p=0.002) and activities at work and school (1293 ± 940 min/week vs. 792 ± 794 min/week, p<0.0001).

**Table 3:** **Start to Run group**: **changes in physical activity after six weeks**

**Outcome measures**	**Start to Run group (n=123)**		
	**Baseline**	**After six weeks**	**P**^**a**^
Dutch physical activity guidelines (%)			
Compliance with DNHPA	43.9	74.8	<0.0001*
Compliance with Fit-norm	53.7	87.0	<0.0001*
Compliance with Combi-norm	57.7	91.1	<0.0001*
Physical activity by intensity, mean ± SD (min/week)			
Light-intensity activities	1827 ± 1192	1423 ± 1296	0.002*
Moderate-intensity activities	209 ± 462	163 ± 253	0.21
Vigorous-intensity activities	200 ± 205	410 ± 298	<0.0001*
Physical activity by domain, mean ± SD (min/week)			
Commuting activities	70 ± 110	98 ± 155	0.01*
Leisure-time activities	240 ± 268	301 ± 343	0.02*
Sports activities	101 ± 143	243 ± 173	<0.0001*
Household activities	563 ± 759	614 ± 887	0.43
Activities at work and school	1293 ± 940	792 ± 794	<0.0001*
Total time spent in physical activity, mean ± SD (min/week)	2237 ± 1183	1996 ± 1451	0.08

^a^P-value for difference in physical activity within the Start to Run group.

*Significant (p<0.05) change in physical activity after six weeks within the Start to Run group.

#### Changes in physical activity after six months: comparisons within groups

In Table 4, physical activity outcome measures are presented for both the Start to Run group and control group at baseline and after six months. In the Start to Run group, the percentage of people who met the DNHPA (48.0% vs. 64.0%, p=0.004), Fit-norm (56.0% vs. 82.0%, p<0.0001), and Combi-norm (58.0% vs. 84.0%, p<0.0001) increased significantly between baseline and six months. These changes were accompanied by a significant increase in the total time spent in physical activity (2265 ± 1251 min/week vs. 2536 ± 1210 min/week, p=0.04). Also, significant changes in physical activity behavior were observed within physical activity intensity categories and domains, i.e. after six months, the Start to Run participants spent more time in vigorous-intensity activities (238 ± 250 min/week vs. 382 ± 306 min/week, p<0.0001), commuting activities (88 ± 137 min/week vs. 132 ± 181 min/week, p=0.006) and sports activities (126 ± 166 min/week vs. 225 ± 182 min/week, p<0.0001). In contrast, the control group participants did not significantly change their physical activity behavior between baseline and six months.

**Table 4:** **Changes in physical activity after six months**: **comparisons within groups**

**Outcome measures**	**Start to Run group (n=100)**			**Control group (n=100)**		
	**Baseline**	**After six months**	**P**^**a**^	**Baseline**	**After six months**	**P**^**b**^
Dutch physical activity guidelines (%)						
Compliance with DNHPA	48.0	64.0	0.004*	59.0	62.0	0.68
Compliance with Fit-norm	56.0	82.0	<0.0001*	55.0	57.0	0.83
Compliance with Combi-norm	58.0	84.0	<0.0001*	65.0	73.0	0.10
Physical activity by intensity, mean ± SD (min/week)						
Light-intensity activities	1814 ± 1224	1947 ± 1043	0.31	1958 ± 1263	1972 ± 1181	0.90
Moderate-intensity activities	213 ± 453	206 ± 369	0.88	406 ± 596	450 ± 740	0.47
Vigorous-intensity activities	238 ± 250	382 ± 306	<0.0001*	253 ± 337	238 ± 286	0.60
Physical activity by domain, mean ± SD (min/week)						
Commuting activities	88 ± 137	132 ± 181	0.006*	117 ± 251	124 ± 215	0.80
Leisure-time activities	257 ± 296	276 ± 358	0.48	328 ± 407	325 ± 515	0.91
Sports activities	126 ± 166	225 ± 182	<0.0001*	107 ± 147	108 ± 142	0.96
Household activities	552 ± 780	585 ± 597	0.66	919 ± 968	807 ± 856	0.15
Activities at work and school	1309 ± 935	1381 ± 864	0.49	1182 ± 951	1322 ± 887	0.11
Total time spent in physical activity, mean ± SD (min/week)	2265 ± 1251	2536 ± 1210	0.04*	2616 ± 1356	2660 ± 1126	0.73

^a^P-value for difference in physical activity within the Start to Run group.

^b^P-value for difference in physical activity within the control group.

*Significant (p<0.05) change in physical activity after six months within the Start to Run group.

#### Changes in physical activity after six months: comparisons between groups

The results of the multiple linear and logistic regression analyses are presented in Table 5. After six months, significantly more Start to Run participants compared with control group participants were meeting the Fit-norm (OR=5.1; 95% CI: 2.3-11.1, p<0.001) and Combi-norm (OR=3.3; 95% CI: 1.4-7.7, p=0.006). In addition, significant effects were found in favor of the Start to Run group concerning physical activity intensity categories and domains: after six months, the Start to Run participants were spending more time in vigorous-intensity activities (an average of 152 min/week more: b=152; 95% CI: 80-223, p<0.001) and sports activities (an average of 107 min/week more: b=107; 95% CI: 69-145, p<0.001) compared with controls. For the remaining physical activity outcome measures, no significant differences were found between groups.

**Table 5:** **Changes in physical activity after six months**: **comparisons between groups**

**Dichotomous outcome measures**	**OR (group variable)**^**a**^	**95% CI**	**P (group variable)**
Dutch physical activity guidelines			
Compliance with DNHPA	1.5	0.8-3.0	0.22
Compliance with Fit-norm	5.1	2.3-11.1	<0.001*
Compliance with Combi-norm	3.3	1.4-7.7	0.006*
**Continuous outcome measures**	**b-coefficient (group variable)**^**a,b**^	**95% CI**	**P (group variable)**
Physical activity by intensity			
Light-intensity activities	38	−234-311	0.78
Moderate-intensity activities	−126	−265-12	0.07
Vigorous-intensity activities	152	80-223	<0.001*
Physical activity by domain			
Commuting activities	22	−27-71	0.37
Leisure-time activities	19	−63-101	0.65
Sports activities	107	69-145	<0.001*
Household activities	−45	−220-131	0.62
Activities at work and school	5	−216-226	0.96
Total time spent in physical activity	20	−274-313	0.90

^a^Multiple (linear or logistic) regression analyses were conducted with physical activity level at six months as the dependent variable, and group (Start to Run group versus control group, with the control group as the reference category) as the independent variable. Adjustments were made for baseline physical activity levels.

^b^Unstandardized regression coefficient.

*Significant (p<0.05) difference in physical activity between groups.

## Discussion

The aim of this study was to assess the effectiveness of Start to Run, a 6-week training program for novice runners, on increasing health-enhancing physical activity, both in the short- and longer-term. In the Start to Run group, short- and longer-term beneficial within group effects were observed. In the control group, however, there were no significant changes in physical activity behavior within a period of six months. When comparing results between groups, the Start to Run program produced significant positive changes in health-enhancing physical activity levels: after six months, significantly more Start to Run participants compared with control group participants were meeting the Fit-norm and Combi-norm. The differences in the amount of physical activity between groups in favor of the Start to Run group could be explained by an increase in the time spent in vigorous-intensity activities (physical activity intensity category) and sports activities (physical activity domain).

As running is a vigorous-intensity sports activity, these results are not surprising. This is especially true for the assessment after six weeks. More interesting is the fact that these outcome measures were also positively affected at the six months assessment. Considering the higher levels of vigorous-intensity physical activity and sports activity, the results suggest that most Start to Run participants were still running even 4.5 months after cessation of the Start to Run training program. Some additional results, not presented in the results section, confirm that this was indeed the case: at the six months assessment, running behavior was also directly assessed by a single question: “Are you (still) running at this moment?” The results of this question showed that 69.0% of the Start to Run participants was still performing running activities [see Additional file 1 - Additional results evaluation Start to Run program]. Based on these findings, it seems that Start to Run can recruit people that are insufficiently active; motivate them to take up running; and also frequently and long enough to meet levels of health-enhancing physical activity (as measured by the Fit-norm and Combi-norm). Consequently, Start to Run can positively contribute to improving health of participants.

To sustain health benefits, however, it is important that this running behavior is maintained, i.e. that the Start to Run participants continue to run on a regular basis. Often maintenance is defined as implementing behavior change for at least six months after cessation of intervention [33]. Since the last assessment of physical activity was 4.5 months after cessation of the Start to Run training program, it is difficult to ascertain whether sustained changes in physical activity behavior have been reached according to this definition of maintenance. Others, however, do not define maintenance as sustaining behavior change over a specified period of time. Rothman (2000), for example, rather looks at the processes that govern behavioral maintenance and he argues that people will maintain a change in behavior only if they are satisfied with the new behavior [34]. The Start to Run participants gave the overall training program a rating of 8.2 (scale 0-10; 0 being very poor and 10 being excellent) [see Additional file 1 - Additional results evaluation Start to Run program]. Moreover, the fact that most Start to Run participants were still running 4.5 months after cessation of the Start to Run training program, may on its own indicate that they were satisfied with their new running behavior and thus will continue running. Nonetheless, definite conclusions cannot be drawn and follow-up assessments over longer periods of time are necessary to establish if the Start to Run participants continue their newly acquired physical activity behavior.

With regard to maintaining physical activity levels, the organized sports sector itself may play an important role. In this sector, physical activity opportunities are provided on a continuous basis (i.e. people can play sports on a weekly basis at a sports club). When first providing an easily accessible sporting program, like Start to Run, the next step, i.e. participation in organized sports on a continuous basis, may be facilitated. After completing the program, the Start to Run participants were stimulated to continue running as a member of a local athletics club or the Dutch Athletics Organization. At the six months assessment, 41.0% of the Start to Run participants reported that they became (and still were) a member of a local athletics club or the Dutch Athletics Organization, as a result of participation in the Start to Run training program [see Additional file 1 - Additional results evaluation Start to Run program]. These data suggest that an easily accessible sporting program, like Start to Run, may indeed facilitate participation in organized sports. The role of the organized sports sector in both increasing and maintaining health-enhancing physical activity levels should therefore be further explored.

Next to significant increases in vigorous-intensity physical activity and sports activity, the study had some other interesting findings: after six weeks, the Start to Run participants were spending significantly more time in commuting activities and leisure-time activities. These results suggest that Start to Run may have led to increases in physical activity in these domains. However, in the same period, there was also a significant decrease in the time spent in work and school activities and, consequently, light-intensity activities. These results indicate that, at six weeks, physical activity levels may have been influenced by other factors, like weather conditions, season and/or holidays. The influence of these factors on commuting activities, leisure-time activities and activities at work and school seems plausible, since no effects were found on these outcome measures at the six months assessment when compared with the control group. Yet, without an assessment of the control group at six weeks, some uncertainty remains.

Another interesting finding is that Start to Run did not directly affect the total time spent in physical activity. Despite no significant increases in the total time spent in physical activity, additional health benefits are obtained due to participation in Start to Run: as mentioned before, the increases in sports activity/vigorous-intensity physical activity were substantial, and resulted in more Start to Run participants meeting minimum recommended amounts of vigorous-intensity physical activity for health benefits. Also, there is evidence that vigorous-intensity physical activities, like running, lead to even greater improvements in aerobic fitness and greater reductions in cardiovascular disease and mortality risk than moderate- or light-intensity physical activities, which is independent of their contribution to energy expenditure [35-37].

To our knowledge, this is the first study evaluating the effectiveness of a training program aimed at novice runners on increasing health-enhancing physical activity. In general, there is a lack of research and evaluation of activities conducted in sports settings, especially of controlled study designs incorporating both the short- and longer-term effects [27,28]. Therefore, it is difficult to compare these results with those of previous studies. Most comparable studies would be physical activity intervention studies, and a lot of research has been done in this area [e.g. [38,39]: some physical activity interventions that prescribed running positively affected physical activity behavior of participants. However, comparability is still limited, as these physical activity interventions did not focus on running per se, were often multi-component, took place in non-sports settings and used different outcome measures.

There are some limitations to this study that should be mentioned. First of all, the design of the study does not allow drawing any conclusions on which specific aspect of the Start to Run program (e.g. group sessions, individual sessions, test run) is most important for increasing (and continuing) physical activity. Moreover, participants’ compliance with the different program components was not measured, making it even more difficult to disentangle the most effective program parts. Second, in this study, a self-report measure of physical activity was used. Despite their common use, there are several limitations of self-report tools, including inaccurate recall of the frequency, duration and intensity of physical activity, problems with question comprehension and interpretation, and social desirability bias which can lead to over-reporting of physical activity [40]. However, any inaccuracies are assumed to be random and among both groups. It is therefore unlikely that these potential sources of bias explain the differences in physical activity between the Start to Run group and control group. Self-report measures have the advantage that they are able to collect data from a large number of people at low costs. The SQUASH questionnaire itself has some distinct advantages compared with other physical activity questionnaires: it is short, quick to fill in (3-5 minutes), it measures the amount of physical activity (separately) for five different domains and provides the opportunity to estimate compliance with physical activity guidelines. An alternative to self-report measures is to use more objective instruments to measure physical activity, like accelerometers and heart rate monitors. Compared with self-report measures, objective instruments are more expensive and logistically more difficult to administer on a large scale. For these reasons, it was decided to use a self-report measure. Nonetheless, it would be interesting to see if the results of this study could be replicated with such an objective measure. Third, due to the voluntary nature of participation in the Start to Run training program, the possibility of selection bias cannot be entirely excluded. It could be that people who registered for Start to Run were already highly motivated to increase physical activity levels. Therefore, the findings of this study may not pertain to inactive individuals, i.e. the ones who are often less motivated to increase physical activity levels. On the other hand, also a large group of people who did not meet physical activity guidelines was attracted by the Start to Run training program (i.e. almost half of the Start to Run participants), which may indicate that the program is also suited for this population group. Although this voluntary participation into Start to Run might have caused selection bias, it is a strength of the study as well. First of all, behavior was not forced. Next to that, the study population of Start to Run was a sample of the actual Start to Run population. The Start to Run participants in this study had a mean age of 40 years and the percentage of women was 70.0%. Demographic data collected by the Dutch Athletics Organization of the entire Start to Run population in March 2009 (n=4230) show that the study sample is representative for the entire Start to Run population with regard to age and sex: the average age of the entire Start to Run population was also 40 years and 77.8% of participants was female. Thus, the study was performed in a generalizable group. Moreover, since the study was performed in a real-world setting, namely the sports setting, results are directly transferable into practice. Finally, in this study, it was not possible to ascertain why more than half of the Start to Run participants dropped out of the study between the baseline and six months assessment. It is very difficult to determine why participants did not fill in this questionnaire, because no follow-up data were available of these persons. There could be cases that did not respond to the invitation to fill in this questionnaire because they stopped running (e.g. due to an injury or a bad running experience). Given the very low drop-out rate of the Start to Run training program (according to the Dutch Athletics Organization, only 2.2% of the participants dropped out of the Start to Run training program) and the (already) relatively high drop-out in this the study after six weeks, this seems not a plausible explanation. With regard to baseline characteristics, non-respondents were somewhat younger and more likely to be female. There were, however, no significant differences in baseline physical activity levels between respondents and non-respondents. Therefore, the most likely explanation for the non-response is that participants were not motivated to participate in a scientific study and filling in a questionnaire. Furthermore, since no differences in baseline physical activity levels were found between respondents and non-respondents, it is unlikely that these losses to follow-up influenced study results substantially.

## Conclusions

Considering the above-mentioned limitations, this study does add to the knowledge base concerning the effectiveness of programs initiated in sports settings. The results indicate that an easily accessible program, like Start to Run, organized by a sporting organization, can positively influence levels of health-enhancing physical activity of participants, both in the short- and longer-term. Consequently, Start to Run can lead to tangible health benefits among its participants. Based on these results, the use of the organized sports sector as a setting to promote health-enhancing physical activity seems promising. However, further research is needed to establish maintenance of physical activity behavior and generalizability of these results to other (easily accessible) sporting programs. Moreover, the role of the organized sports sector in maintaining health-enhancing physical activity levels should be further explored. In future studies, it is also recommended to include more in-depth analyses. It is, for example, important to investigate which population groups benefit most from a program like Start to Run (e.g. men vs. women, young adults vs. older adults, obese vs. non-obese people) and to establish the relative effectiveness of program parts. Research in the area of effectiveness of sporting programs in increasing health-enhancing physical activity is still lacking. These data will hopefully encourage policy makers and sporting organizations to both develop and rigorously evaluate easily accessible sporting programs. In this way, more knowledge about the role of the organized sports sector in both promoting and maintaining health-enhancing physical activity can be acquired.

## Abbreviations

ACSM: American College of Sports Medicine; AHA: American Heart Association; DNHPA: Dutch Norm for Health-enhancing Physical Activity; MET: METabolic equivalent; NAPSE: National Action Plan for Sport and Exercise; NIVEL: Netherlands Institute for Health Services Research; SQUASH: Short QUestionnaire to ASsess Health-enhancing physical activity.

## Competing interests

The authors declare that they have no competing interests.

## Authors’ contributions

LO contributed to the design of the study, participated in the data collection process, performed data analysis, and drafted the manuscript. CV and DHB contributed to the design of the study, advised on the analytical approach, and reviewed and commented on the manuscript. All authors read and approved the final manuscript.

## Pre-publication history

The pre-publication history for this paper can be accessed here:

http://www.biomedcentral.com/1471-2458/13/697/prepub

## Supplementary Material

Additional file 1 **Additional results evaluation Start to Run program.** In the additional file, results can be found concerning the evaluation of the Start to Run program that are not shown in the results section of the article. Click here for file

## References

[B1] Gill JM Cooper AR Physical activity and prevention of type 2 diabetes mellitus Sports Med 2008 38 807 824 10.2165/00007256-200838100-00002 18803434

[B2] Jakicic JM The effect of physical activity on body weight Obesity (Silver Spring) 2009 17 Suppl 3 S34 S38 19927144 10.1038/oby.2009.386

[B3] Schmitt NM Schmitt J Doren M The role of physical activity in the prevention of osteoporosis in postmenopausal women-An update Maturitas 2009 63 34 38 10.1016/j.maturitas.2009.03.002 19356867

[B4] Sherrington C Whitney JC Lord SR Herbert RD Cumming RG Close JC Effective exercise for the prevention of falls: a systematic review and meta-analysis J Am Geriatr Soc 2008 56 2234 2243 10.1111/j.1532-5415.2008.02014.x 19093923

[B5] Sofi F Capalbo A Cesari F Abbate R Gensini GF Physical activity during leisure time and primary prevention of coronary heart disease: an updated meta-analysis of cohort studies Eur J Cardiovasc Prev Rehabil 2008 15 247 257 10.1097/HJR.0b013e3282f232ac 18525378

[B6] Thune I Furberg AS Physical activity and cancer risk: dose–response and cancer, all sites and site-specific Med Sci Sports Exerc 2001 33 S530 S550 10.1097/00005768-200106001-00025 11427781

[B7] Wendel-Vos GC Schuit AJ Feskens EJ Boshuizen HC Verschuren WM Saris WH Kromhout D Physical activity and stroke. A meta-analysis of observational data Int J Epidemiol 2004 33 787 798 10.1093/ije/dyh168 15166195

[B8] Bize R Johnson JA Plotnikoff RC Physical activity level and health-related quality of life in the general adult population: a systematic review Prev Med 2007 45 401 415 10.1016/j.ypmed.2007.07.017 17707498

[B9] Strohle A Physical activity, exercise, depression and anxiety disorders J Neural Transm 2009 116 777 784 10.1007/s00702-008-0092-x 18726137

[B10] Haskell WL Lee IM Pate RR Powell KE Blair SN Franklin BA Macera CA Heath GW Thompson PD Bauman A Physical activity and public health: updated recommendation for adults from the American College of Sports Medicine and the American Heart Association Med Sci Sports Exerc 2007 39 1423 1434 10.1249/mss.0b013e3180616b27 17762377

[B11] Hildebrandt VH Chorus AMJ Stubbe JH Trend report of physical activity and health 2008/2009 2010 Leiden, The Netherlands: TNO Quality of Life

[B12] Eyler AA Brownson RC Donatelle RJ King AC Brown D Sallis JF Physical activity social support and middle- and older-aged minority women: results from a US survey Soc Sci Med 1999 49 781 789 10.1016/S0277-9536(99)00137-9 10459889

[B13] Sallis JF Hovell MF Hofstetter CR Predictors of adoption and maintenance of vigorous physical activity in men and women Prev Med 1992 21 237 251 10.1016/0091-7435(92)90022-A 1579558

[B14] Wendel-Vos W Droomers M Kremers S Brug J van Lenthe F Potential environmental determinants of physical activity in adults: a systematic review Obes Rev 2007 8 425 440 10.1111/j.1467-789X.2007.00370.x 17716300

[B15] European Commission Sport and Physical Activity. Special Eurobarometer 334/Wave 72.3 2010 Brussels, Belgium: TNS Opinion & Social

[B16] Dobbinson SJ Hayman JA Livingston PM Prevalence of health promotion policies in sports clubs in Victoria, Australia Health Promot Int 2006 21 121 129 10.1093/heapro/dak001 16403799

[B17] Dutch Ministry of Health, Welfare and Sport Time for sport: exercise, participate, perform 2005 The Hague, The Netherlands: Dutch Ministry of Health, Welfare and Sport

[B18] Eime RM Payne WR Harvey JT Making sporting clubs healthy and welcoming environments: a strategy to increase participation J Sci Med Sport 2008 11 146 154 10.1016/j.jsams.2006.12.121 17544843

[B19] Kokko S Kannas L Villberg J The health promoting sports club in Finland–a challenge for the settings-based approach Health Promot Int 2006 21 219 229 10.1093/heapro/dal013 16684782

[B20] Whitelaw S Baxendale A Bryce C MacHardy L Young I Witney E 'Settings' based health promotion: a review Health Promot Int 2001 16 339 353 10.1093/heapro/16.4.339 11733453

[B21] World Health Organization (WHO) Ottawa Charter for Health Promotion 1986 Geneva: WHO

[B22] Buist I Bredeweg SW van Mechelen W Lemmink KA Pepping GJ Diercks RL No effect of a graded training program on the number of running-related injuries in novice runners: a randomized controlled trial Am J Sports Med 2008 36 33 39 17940147 10.1177/0363546507307505

[B23] Chakravarty EF Hubert HB Lingala VB Fries JF Reduced disability and mortality among aging runners: a 21-year longitudinal study Arch Intern Med 2008 168 1638 1646 10.1001/archinte.168.15.1638 18695077 PMC3175643

[B24] Koplan JP Powell KE Sikes RK Shirley RW Campbell CC An epidemiologic study of the benefits and risks of running JAMA 1982 248 3118 3121 10.1001/jama.1982.03330230030026 7143687

[B25] Taunton JE Ryan MB Clement DB McKenzie DC Lloyd-Smith DR Zumbo BD A prospective study of running injuries: the Vancouver Sun Run "In Training" clinics Br J Sports Med 2003 37 239 244 10.1136/bjsm.37.3.239 12782549 PMC1724633

[B26] Williams PT A cohort study of incident hypertension in relation to changes in vigorous physical activity in men and women J Hypertens 2008 26 1085 1093 10.1097/HJH.0b013e3282fb81dc 18475145 PMC2828465

[B27] Priest N Armstrong R Doyle J Waters E Interventions implemented through sporting organisations for increasing participation in sport Cochrane Database Syst Rev 2008 3 Art. No.: CD004812. 10.1002/14651858.CD004812.pub3 PMC13273799 18646112

[B28] Priest N Armstrong R Doyle J Waters E Policy interventions implemented through sporting organisations for promoting healthy behaviour change Cochrane Database Syst Rev 2008 3 Art. No.: CD004809. 10.1002/14651858.CD004809.pub3 PMC6464902 18646111

[B29] Reitsma-van Rooijen M de Jong JD Health Care Consumer Panel: basic report with information about the panel, 2009 2009 Utrecht, The Netherlands: Netherlands Institute for Health Services Research (NIVEL)

[B30] Bauman A Bull F Chey T Craig CL Ainsworth BE Sallis JF Bowles HR Hagstromer M Sjostrom M Pratt M The International Prevalence Study on Physical Activity: results from 20 countries Int J Behav Nutr Phys Act 2009 6 21 10.1186/1479-5868-6-21 19335883 PMC2674408

[B31] Wendel-Vos GC Schuit AJ Saris WH Kromhout D Reproducibility and relative validity of the short questionnaire to assess health-enhancing physical activity J Clin Epidemiol 2003 56 1163 1169 10.1016/S0895-4356(03)00220-8 14680666

[B32] Ainsworth BE Haskell WL Whitt MC Irwin ML Swartz AM Strath SJ O'Brien WL Bassett DR Jr Schmitz KH Emplaincourt PO Jacobs DR Jr Leon AS Compendium of physical activities: an update of activity codes and MET intensities Med Sci Sports Exerc 2000 32 S498 S504 10.1097/00005768-200009001-00009 10993420

[B33] Marcus BH Dubbert PM Forsyth LH McKenzie TL Stone EJ Dunn AL Blair SN Physical activity behavior change: issues in adoption and maintenance Health Psychol 2000 19 32 41 10709946 10.1037/0278-6133.19.suppl1.32

[B34] Rothman AJ Toward a theory-based analysis of behavioral maintenance Health Psychol 2000 19 64 69 10709949 10.1037/0278-6133.19.suppl1.64

[B35] Lee IM Paffenbarger RS Jr Associations of light, moderate, and vigorous intensity physical activity with longevity. The Harvard Alumni Health Study Am J Epidemiol 2000 151 293 299 10.1093/oxfordjournals.aje.a010205 10670554

[B36] Swain DP Moderate or vigorous intensity exercise: which is better for improving aerobic fitness? Prev Cardiol 2005 8 55 58 10.1111/j.1520-037X.2005.02791.x 15722695

[B37] Swain DP Franklin BA Comparison of cardioprotective benefits of vigorous versus moderate intensity aerobic exercise Am J Cardiol 2006 97 141 147 10.1016/j.amjcard.2005.07.130 16377300

[B38] Foster C Hillsdon M Thorogood M Interventions for promoting physical activity Cochrane Database Syst Rev 2005 1 Art. No.: CD003180. 10.1002/14651858.CD003180.pub2 PMC4164373 15674903

[B39] Marcus BH Williams DM Dubbert PM Sallis JF King AC Yancey AK Franklin BA Buchner D Daniels SR Claytor RP Physical activity intervention studies: what we know and what we need to know: a scientific statement from the American Heart Association Council on Nutrition, Physical Activity, and Metabolism (Subcommittee on Physical Activity); Council on Cardiovascular Disease in the Young; and the Interdisciplinary Working Group on Quality of Care and Outcomes Research Circulation 2006 114 2739 2752 10.1161/CIRCULATIONAHA.106.179683 17145995

[B40] Sallis JF Saelens BE Assessment of physical activity by self-report: status, limitations, and future directions Res Q Exerc Sport 2000 71 S1 14 10.1080/02701367.2000.10608875 10925819

