# Supplementary material for: Effectiveness of Start to Run, a 6-week training program for novice runners, on increasing health-enhancing physical activity: a controlled study
Source: BMC Public Health. 2013 Jul 31;13:697. doi: 10.1186/1471-2458-13-697 (PMC3735486; doi:10.1186/1471-2458-13-697)
Supplement: Additional file 1 — Additional results evaluation Start to Run program. In the additional file, results can be found concerning the evaluation of the Start to Run program that are not shown in the results section of the article. [file 1471-2458-13-697-S1.doc]

**Additional results evaluation Start to Run program**

Table 1. Start to Run group: running behavior and membership at the six months assessment

|  | **Start to Run group (n=100)** |
| --- | --- |
| Percentage of participants that is (still) running | 69.0% |
| Percentage of participants that became (and still is) a member of a local athletics club or the Dutch Athletics Organization due to participation in Start to Run | 41.0% |

Table 2. Overall rating Start to Run training program

|  | **Start to Run group (n=123)** |
| --- | --- |
| Rating (scale 0-10; 0 being very poor and 10 being excellent),  mean ± SD | 8.2 ± 1.2 |
